# Glial GLT-1 blockade in infralimbic cortex as a new strategy to evoke rapid antidepressant-like effects in rats

**DOI:** 10.1038/tp.2017.7

**Published:** 2017-02-21

**Authors:** J Gasull-Camós, M Tarrés-Gatius, F Artigas, A Castañé

**Affiliations:** 1 Department of Neurochemistry and Neuropharmacology, CSIC-Institut d'Investigacions Biomèdiques de Barcelona, Barcelona, Spain; 2 Centro de Investigación Biomédica en Red de Salud Mental, Instituto de Salud Carlos III, Madrid, Spain; 3 Institut d'Investigacions Biomèdiques August Pi i Sunyer, Barcelona, Spain

## Abstract

Ketamine and deep brain stimulation produce rapid antidepressant effects in humans and rodents. An increased AMPA receptor (AMPA-R) signaling in medial prefrontal cortex (mPFC) has been suggested to mediate these responses. However, little research has addressed the direct effects of enhancing glutamate tone or AMPA-R stimulation in mPFC subdivisions. The current study investigates the behavioral and neurochemical consequences of glutamate transporter-1 (GLT-1) blockade or s-AMPA microinfusion in the infralimbic (IL) and prelimbic (PrL) cortex. Owing to the connectivity between the mPFC and raphe nuclei, the role of serotonin is also explored. The bilateral microinfusion of the depolarizing agent veratridine into IL -but not PrL- of rats evoked immediate antidepressant-like responses. The same regional selectivity was observed after microinfusion of dihydrokainic acid (DHK), a selective inhibitor of GLT-1, present in astrocytes. The DHK-evoked antidepressant-like responses appear to be mediated by an AMPA-R-driven enhancement of serotonergic activity, as (i) they were prevented by NBQX 2,3-dioxo-6-nitro-1,2,3,4-tetrahydrobenzo[f]quinoxaline-7-sulfonamide disodium salt) and mimicked by s-AMPA; (ii) DHK and s-AMPA elevated similarly extracellular glutamate in IL and PrL, although extracellular 5-HT and *c-fos* expression in the midbrain dorsal raphe increased only when these agents were applied in IL; and (iii) DHK antidepressant-like responses were prevented by 5-HT synthesis inhibition and mimicked by citalopram microinfusion in IL. These results indicate that an acute increase of glutamatergic neurotransmission selectively in IL triggers immediate antidepressant-like responses in rats, likely mediated by the activation of IL–raphe pathways, which then results in a fast increase of serotonergic activity.

## Introduction

Major depressive disorder is a leading cause of disability worldwide with a high socioeconomic impact.^1^ Standard treatments, based on serotonin (5-HT) and/or norepinephrine reuptake inhibition, show slow onset of clinical action and limited efficacy, which results in a high percentage of chronic or recurrent patients.^2, 3^ The non-competitive *N*-methyl-d-aspartate receptor antagonist ketamine and deep brain stimulation (DBS) overcome some of these limitations and evoke a rapid and long-lasting clinical improvement in treatment-resistant patients.^4, 5, 6, 7^ Currently, research efforts are focused on identifying the mechanism procuring these rapid antidepressant actions.

The prefrontal cortex (PFC) has a crucial role in the pathophysiology and treatment of major depressive disorder. Early neuroimaging studies reported on a reduced energy metabolism in the subgenual anterior cingulate cortex of major depressive disorder patients, associated with a glial loss.^8, 9^ Further studies indicated an increased activity of the adjacent Brodmann area 25, which was normalized after effective treatment including DBS.^4, 10, 11, 12, 13^ In some contrast to the latter observations, sub-anesthetic doses of ketamine increased glucose metabolism in this area in healthy volunteers.^14, 15, 16^

In rats, the most ventral part of the medial PFC (mPFC), namely the infralimbic cortex (IL), has emerged as an important hub for emotional control.^17, 18, 19^ Indeed, DBS of the IL but not prelimbic cortex (PrL) induced robust antidepressant-like responses in rats.^20^ Likewise, local IL ketamine infusion evoked rapid and long-lasting effects, which were mimicked by the optogenetic stimulation of IL^21^ suggesting that ketamine's antidepressant action is mediated by an increased excitatory neurotransmission in this area. In agreement, ketamine has been shown to increase glutamate release in rat PFC.^22^ Moreover, ketamine and DBS induce antidepressant-like responses that require the activation of AMPA-R.^20, 23^

Here we further assess the role of excitatory neurotransmission in dorsal (PrL) and ventral (IL) mPFC subdivisions in the control of emotional states. For this purpose, we investigated the behavioral and neurochemical consequences of blocking the glutamate transporter-1 (GLT-1), mainly localized in astrocytes and mainly responsible for cortical glutamate reuptake.^24, 25^ Moreover, owing to the reciprocal connectivity and mutual control between mPFC and the raphe nuclei,^26, 27, 28^ we investigated the contribution of serotonin neurotransmission on these effects.

## Materials and methods

### Subjects

Male Wistar rats (Charles River, Lyon, France) weighing 280–330 g at the time of surgery were used. The rats were maintained in a controlled environment (12 h light/dark, 22±1 ºC) with *ad libitum* access to food and water. Before surgery, the rats were acclimatized to the housing conditions for at least 7 days and were daily handled. After surgery, the rats were singly housed and randomly assigned to treatment. All the experimental procedures were conducted in accordance with national (Royal Decree 53/2013) and European legislation (Directive 2010/63/EU, on the protection of animals used for scientific purposes, 22 September 2010), and were approved by the Institutional Animal Care and Use Committee of the University of Barcelona.

### Drugs

Veratridine, dihydrokainic acid (DHK), 2,3-dioxo-6-nitro-1,2,3,4-tetrahydrobenzo[f]quinoxaline-7-sulfonamide disodium salt (NBQX), (*S*)-α-Amino-3-hydroxy-5-methyl-4-isoxazolepropionic acid (s-AMPA) and citalopram hydrobromide were purchased from Tocris (Bristol, UK). 4-chloro-dl-phenylalanine methyl ester hydrochloride (pCPA) was purchased from Sigma-Aldrich (Tres Cantos, Spain).

For intracerebral microinfusion, veratridine was dissolved in 12.5% dimethyl sulfoxide in artificial cerebrospinal fluid and pH adjusted to 6.5; DHK and s-AMPA were dissolved in PBS 10 × as previously reported;^29^ NBQX and citalopram were dissolved in artificial cerebrospinal fluid. The microinfusion volume was 0.5 μl per side in all cases. pCPA was administered intraperitoneally, was daily prepared by dissolving in saline and pH was adjusted to 6.0 with 0.1 m NaOH.^30^

### Surgery and microinfusions

Anesthetized rats (sodium pentobarbital, 60 mg kg^−1^, intraperitoneally) were implanted with stainless steel 22-gauge bilateral guide cannulae (Plastics One, Roanoke, VA, USA) in the cingulate cortex: anteroposterior +3.2; mediolateral ±0.75; dorsoventral −2.4.^31^ The coordinates in mm were taken from bregma and the skull surface. Guide cannulae were fixed with stainless steel screws using dental acrylic. To prevent occlusion, a dummy cannula was inserted into the guide cannula and manipulated daily. This handling decreased the stress associated with the infusion on the testing day.^32^ The rats were allowed 7 days to recover after surgery.

**Figure 1:**
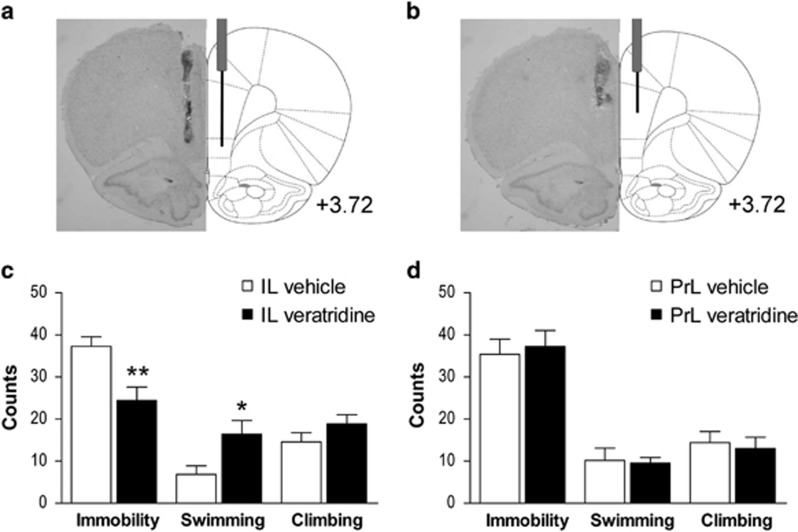
Bilateral microinfusion sites and antidepressant-like action of veratridine on the FST. Neutral red staining of mPFC brain sections at anteroposterior +3.72 from bregma (left) and schematic representation of guide cannula and infusion cannula placement (right) in the (**a**) infralimbic cortex (IL) and (**b**) prelimbic cortex (PrL). (**c**) Bilateral microinfusion of veratridine (50 pmoles) into IL produced a rapid antidepressant-like response, as shown by decreased immobility counts and increased swimming (IL vehicle, *n*=7; IL veratridine, *n*=9). (**d**) Veratridine infusion into PrL produced no behavioral change on the FST (PrL vehicle, *n*=5; PrL veratridine, *n*=7). The values are expressed as mean±s.e.m. *P<0.05 and **P<0.01 versus vehicle-treated rats (Student's *t*-test). FST, forced swimming test; mPFC, medial prefrontal cortex.

The microinfusion cannulae (28 gauge) extended 1.5 or 3 mm beyond the guide cannulae for PrL or IL local administration (dorsoventral: −3.9 or −5.4, respectively). Previous to the tests, three mock infusions were performed. On the testing day, drug or vehicle solutions were bilaterally administered over 1 min through two 50 μl Hamilton syringes connected to the microinfusion cannulae via 0.28 mm ID polyethylene tubing, using an infusion/withdrawal pump (Harvard Apparatus, Holliston, MA, USA). Microinfusion cannulae were left in place for 3 min to allow drug diffusion. Behavioral tests were performed 10 min after drug local administration. In double microinfusion procedures (NBQX and DHK), the second administration occurred 5 min after the first. Figure 1 shows an example of IL (a) and PrL (b) microinfusion sites.

### Behavioral studies

The forced swimming test (FST) was conducted as described previously.^33^ Briefly, a clear methacrylate cylinder (46 × 20 cm) filled with water (24±1 ºC) to a depth of 30 cm was used. Each rat was placed into the cylinder for 15 min in a pretest session where no scoring of behavior was needed. Twenty-four hours later, the rats were exposed to the same conditions during a 5 min test and behavior was video recorded. In a posterior analysis, test recordings were divided into periods of 5 s and the predominant behavior was rated as immobility, swimming or climbing by an experimenter blind to the treatment.

The novelty-suppressed feeding test (NSFT) assesses the rat's aversion to eat in a novel environment. Before the test rats were food restricted (18 g per day, 3 days + 24 h deprivation). On the test day, rats were placed into a novel arena (90 × 90 × 40 cm) for 10 min containing two food pellets in the highly illuminated center (600 lx) and latency to eat was measured, only sniffing was not considered. As a control, homecage food consumption was measured at 5 min and 24 h after test.

Locomotor activity was assessed to ensure that responses on the FST were due to depression-related effects rather than changes in general activity. Measurements were performed in a dimly lighted black open field (35 × 35 cm) and video recorded during 15 min. The distance moved was calculated for each animal using the VideoTrack View Point software (Lyon, France).

### *In vivo* microdialysis studies and tissue 5-HT assessment

Microdialysis experiments were conducted as previously described.^20, 34^ Concentric dialysis probes (1.5 mm membrane length) were implanted in anesthetized rats (sodium pentobarbital, 60 mg kg^−1^, intraperitoneally) unilaterally in the PrL (anteroposterior +3.2; mediolateral −0.6; dorsoventral −4.3) or IL (anteroposterior +3.2; mediolateral −0.6; dorsoventral −5.7). The coordinates were taken from bregma and the skull.^31^ The rats were continuously perfused with artificial cerebrospinal fluid containing 1 μM citalopram at a rate of 1.65 μl/min. A stabilization period of 3 h was used. The effects of vehicle perfusion (10% PBS 10 × in artificial cerebrospinal fluid) and increasing doses of DHK (3 and 10 mm) or s-AMPA (100 μm) were tested at 24 and 48 h after surgery, respectively. Dialysate samples were collected every 25 (DHK) or 35 min (s-AMPA). Neurotransmitter concentrations were determined by high performance liquid chromatography with electrochemical (5-HT) or fluorimetric (glutamate) detection.

The 5-HT depletion was assessed as reported earlier.^35^ Briefly, the mPFC (25–50 mg) and dorsal raphe (DR) (10–15 mg) brain samples were homogenized adding a buffer solution (0.4 m perchloric acid, 0.1% sodium metabisulphite, 0.01% EDTA and 0.1% cysteine; 100 μl buffer per 10 mg of wet tissue). The homogenates were centrifuged (4 °C, 30 min, 12 000 r.p.m.) and the supernatants were filtered (Millex 0.45 μm filters, Merck Millipore, Madrid, Spain) and analyzed by high performance liquid chromatography with electrochemical detection.

### *In situ* hybridization studies

The effects of IL and PrL DHK and s-AMPA on brain *c-fos* mRNA expression were examined by *in situ* hybridization 1 h after treatment, as described previously.^36^ The brain sections (14 μm) were thaw-mounted onto APTS (3-aminopropyltriethoxysilane, Sigma, St Louis, MO, USA)-coated slides and kept at −30 °C. The *c-fos* oligonucleotide probe was complementary to bases 131–178 (GenBank ID: NM_022197) and was labeled with [^33^P]-dATP (>2500 Ci mmol^−1^; DuPont-NEN, Boston, MA, USA) with terminal deoxynucleotidyltransferase (TdT, Calbiochem, La Jolla, CA, USA) and purified with ProbeQuant G-50 Micro Columns (GE Healthcare UK Limited, Buckinghamshire, UK). Hybridized sections were exposed to Biomax MR film (Kodak, Sigma-Aldrich, Madrid, Spain) for 7 days with intensifying screens. Relative optical densities were measured with a computer-assisted image analyzer (MCID, Mering, Germany). Three consecutive brain sections at any level of interest were analyzed for each rat and averaged to obtain individual values.

### Histological verification

At the end of the studies, the rats were killed by sodium pentobarbital overdose and the brains were removed to proceed with the histological verification of the cannulae and probes placement. The brain sections (30 μm) were mounted onto slides and posteriorly stained with neutral red. Rats infused outside PrL or IL were excluded from all analyses.

### Statistical analysis

The number of animals used for each test is reported in the figure legends. The sample sizes were determined based on power analysis and common practice in behavioral, neurochemical (~10 animals per group) and *in situ* hybridization studies (~5 animals per group). The data are expressed as mean±s.e.m. Statistical analysis was carried out using unpaired Student's *t*-test, one-way analysis of variance (ANOVA) followed by Bonferroni *post hoc* comparisons and two-way repeated-measures ANOVA. In the NSFT, we also used nonparametric statistics, the Kaplan–Meier estimator as described previously.^37^ In all cases, the level of significance was set at *P*<0.05.

## Results

### Veratridine microinfusion in IL, but not PrL, induces antidepressant responses on the FST

The bilateral microinfusion of the depolarizing drug veratridine (50 pmoles per side) into IL produced an antidepressant-like response on the FST (Figure 1), reducing immobility (*t*_(14)_=3.120; *P*<0.01) and increasing swimming behavior (*t*_(14)_=2.323; *P*<0.05; Figure 1c). On the contrary, veratridine microinfusion into the PrL produced no significant behavioral responses (Figure 1d).

### DHK microinfusion in IL, but not PrL, induces antidepressant responses on the FST and NSFT

**Figure 2:**
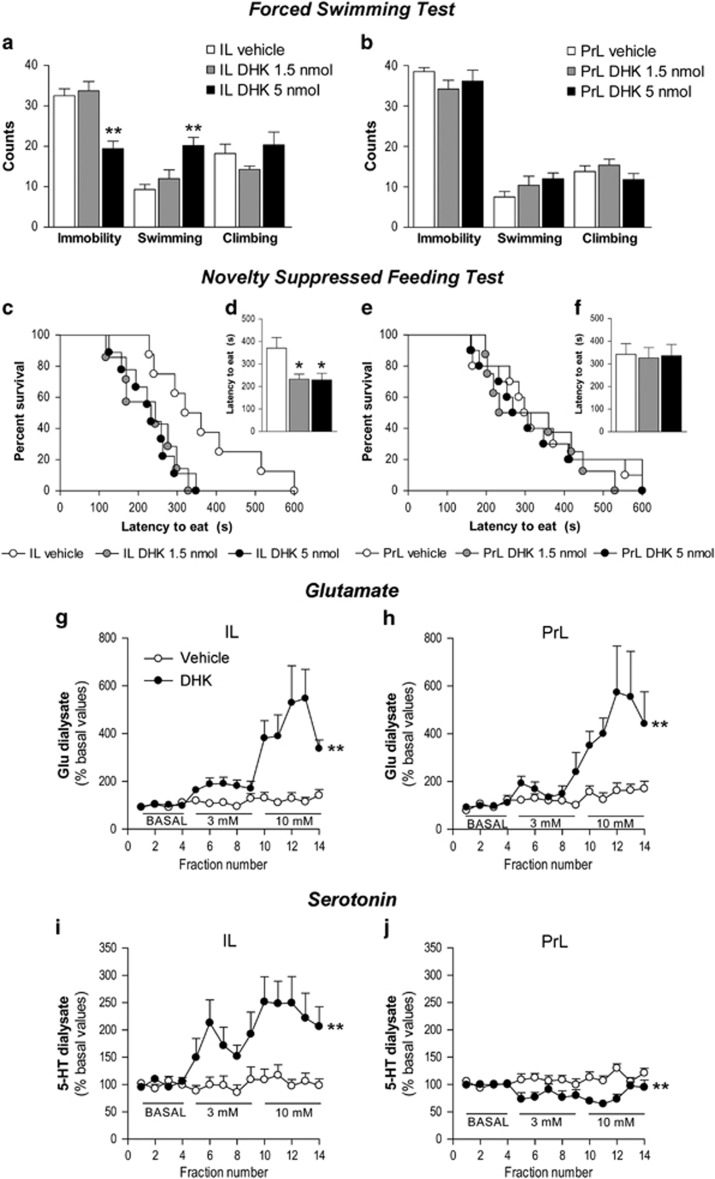
Antidepressant-like action of DHK on the FST and NSFT and neurochemical effects over glutamate and serotonin output. (**a**) Bilateral microinfusion of DHK (5 nmoles) into the IL produced a rapid antidepressant-like response on the FST, as shown by decreased immobility and increased swimming (IL vehicle, *n*=10; IL DHK 1.5 nmoles, *n*=7; IL DHK 5 nmoles, *n*=9). (**b**) PrL DHK infusion produced no behavioral change on the FST (PrL vehicle, *n*=6; PrL DHK 1.5 nmoles, *n*=5; PrL DHK 5 nmoles, *n*=6). IL DHK (1.5 and 5 nmoles) also produced an antidepressant-like response on the NSFT, as shown by (**c**) increased percentage of animals that have eaten in the cumulative survival curve and (**d**) reduced latency to eat (IL vehicle, *n*=8; IL DHK 1.5 nmoles, *n*=9; IL DHK 5 nmoles, *n*=7), whereas (**e** and **f**) PrL DHK produced no behavioral change on the NSFT (PrL vehicle, *n*=10; PrL DHK 1.5 nmoles, *n*=8; PrL DHK 10 nmoles, *n*=10). In microdialysis experiments, DHK dose-dependently increased the extracellular glutamate (Glu) concentration both in (**g**) IL (vehicle; DHK, *n*=10) and (**h**) PrL (vehicle, *n*=10; DHK, *n*=7). Extracellular serotonin (5-HT) concentration (**i**) increased in IL during DHK perfusion (vehicle, *n*=8; DHK, *n*=7), (**j**) whereas small 5-HT decreases were observed during DHK perfusion in PrL (vehicle; DHK, *n*=7). The values are expressed as mean±s.e.m. *P<0.05 and **P<0.01 versus vehicle-treated rats (Bonferroni *post hoc* test). Microdialysis data are expressed as percentages of four basal values. DHK, dihydrokainic acid; FST, forced swimming test; IL, infralimbic cortex; mPFC, medial prefrontal cortex; NSFT, novelty-suppressed feeding test; PrL, prelimbic cortex.

The microinfusion of the GLT-1 inhibitor DHK into IL produced an antidepressant-like response on the FST (Figure 2), reducing the immobility (F_2,25_=16.917; *P*<0.0001) and increasing swimming behavior (F_2,25_=9.990; *P*<0.001). *Post hoc* comparisons revealed significant differences versus controls at 5 but not 1.5 nmoles of DHK (*P*<0.01, in all cases; Figure 2a). A dose of 0.15 nmoles of DHK was also tested with no changes in the immobility time (s) (147.27±12.12) compared with controls (153.45±16.49; *t*_(16)_=0.302; not significant (NS)). The bilateral IL DHK microinfusion produced an antidepressant-like response on the NSFT (Figure 2). Thus, Kaplan–Meier survival analysis showed an effect of treatment (log-rank test, *P*<0.05) and a significant difference between 1.5 and 5 nmoles versus vehicle (*P*<0.05, *P*<0.01, respectively; Figure 2c). Moreover, one-way ANOVA showed a significant effect of treatment on the latency to eat (F_2,23_=5.605; *P*<0.05; Figure 2d). *Post hoc* comparisons showed that both doses of DHK reduced the latency to eat compared with vehicle (*P*<0.05, all cases). Homecage food intake was similar between IL DHK and vehicle groups for the 5 min test (data in grams; vehicle: 0.68±0.12; 1.5 nmoles: 0.84±0.16; 5 nmoles: 0.62±0.13; F_2,23_=0.710; NS) and for the 24 h test data (vehicle: 32.91±0.89; 1.5 nmoles: 32.08±1.32; 5 nmoles: 32.79±1.36; F_2,23_=0.145; NS).

However, DHK microinfusion into the PrL produced no significant behavioral responses on the FST (Figure 2b) and NSFT (Figures 2e and f).

### IL and PrL DHK effects on glutamate and 5-HT output

Baseline concentrations of glutamate (pmol per fraction) in IL and PrL were 11.2±1.3 (*n*=20) and 13.2±1.7 (*n*=17), respectively (NS). DHK perfusion dose-dependently enhanced extracellular glutamate in IL and PrL, with significant effects of the treatment (F_1,18_=14.021; *P*<0.01), fraction (F_13,234_=8.721; *P*<0.0001) and treatment × fraction interaction (F_13,234_=7.057; *P*<0.0001; IL data; Figure 2g, Supplementary Figure S1a) and significant effect of treatment (F_1,15_=12.980; *P*<0.01), fraction (F_13,195_=7.793; *P*<0.0001) and treatment × fraction interaction (F_13,195_=4.575; *P*<0.0001; PrL data; Figure 2h, Supplementary Figure S1b).

Baseline concentrations of 5-HT (fmol per fraction) in IL and PrL were 5.8±1.1 (*n*=14) and 4.4±0.3 (*n*=14), respectively (NS). DHK perfusion into IL markedly increased 5-HT release, with a significant effect of treatment (F_1,13_=10.041; *P*<0.01), fraction (F_13,169_=4.852; *P*<0.0001) and treatment × fraction interaction (F_13,169_=3.952; *P*<0.0001; Figure 2i, Supplementary Figure S1c). Conversely, PrL DHK perfusion decreased extracellular 5-HT, with a significant effect of treatment (F_1,12_ =13.382; *P*<0.01) and treatment × fraction interaction (F_13,156_=3.388; *P*<0.001; Figure 2j, Supplementary Figure S1d).

### Prevention of the antidepressant responses of IL DHK by NBQX pretreatment

**Figure 3:**
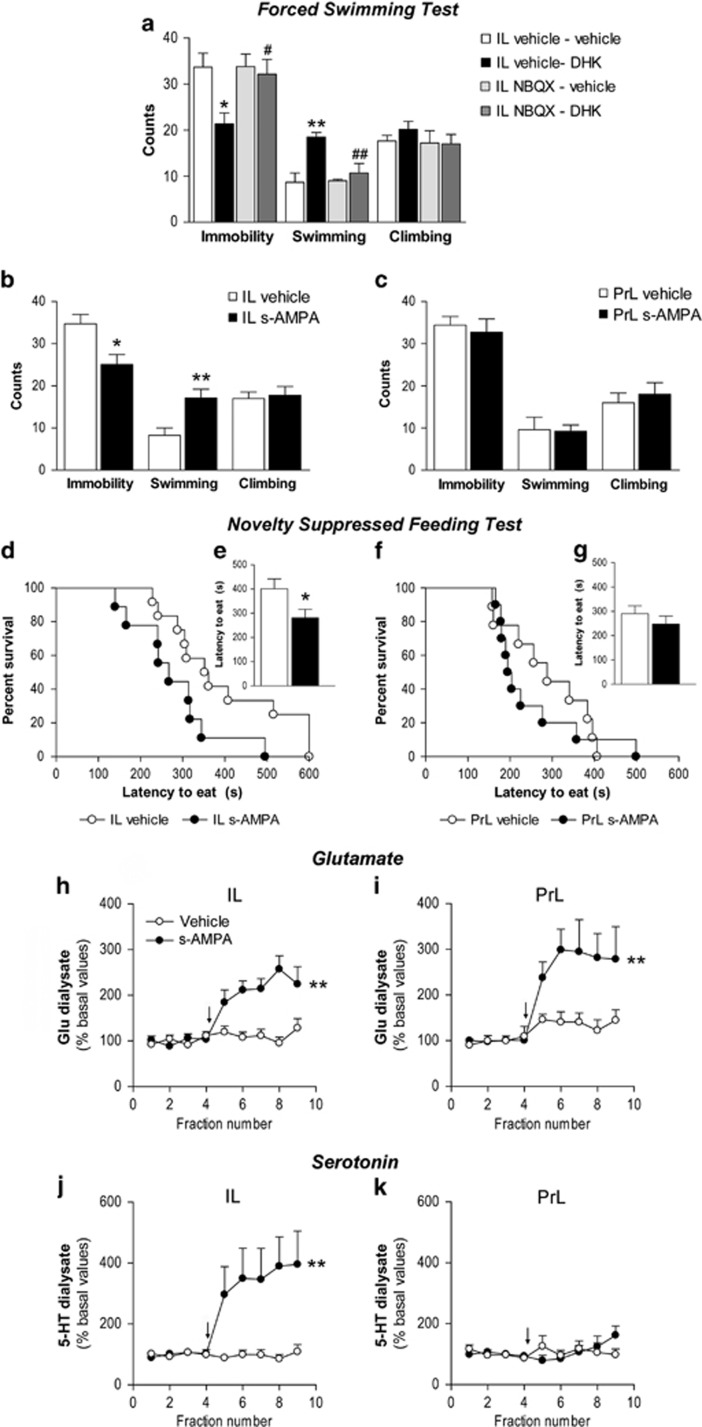
AMPA-R mediates DHK-induced antidepressant-like actions and s-AMPA mimics DHK behavioral and neurochemical effects. (**a**) Effects of NBQX (10 nmoles) and DHK (5 nmoles) double microinfusion into the IL cortex on the FST. The reduction in immobility and increase in swimming counts observed in VEH/DHK group (*n*=6) compared with control (VEH/VEH; *n*=6) was prevented by previous local microinfusion of NBQX (NBQX/DHK; *n*=7). NBQX/VEH group (*n*=5) was similar to controls. (**b**) The bilateral microinfusion of s-AMPA (50 pmoles) into IL produced a rapid antidepressant-like response, as shown by decreased immobility and increased swimming counts (IL vehicle, *n*=7; IL s-AMPA, *n*=9). (**c**) s-AMPA microinfusion into PrL produced no behavioral change on the FST (PrL vehicle, *n*=5; PrL s-AMPA, *n*=5). (**d**) IL s-AMPA also produced an antidepressant-like response on the NSFT, as shown by increased percentage of animals that have eaten in the cumulative survival curve and (**e**) reduced latency to eat (IL vehicle, *n*=12; IL s-AMPA, *n*=9), whereas (**f** and **g**) PrL DHK produced no behavioral change on the NSFT (PrL vehicle, *n*=9; PrL s-AMPA, *n*=10). In microdialysis experiments, s-AMPA increased extracellular glutamate (Glu) concentration both in (**h**) IL (vehicle, *n*=10; s-AMPA, *n*=11) and (**i**) PrL (vehicle, *n*=6; s-AMPA, *n*=9). (**j**) IL s-AMPA increased extracellular serotonin concentration (vehicle, *n*=8; s-AMPA, *n*=7), whereas (**k**) no change was observed in PrL s-AMPA microdialysis (vehicle *n*=7; s-AMPA, *n*=9). The values are expressed as mean±s.e.m. *P<0.05, **P<0.01 versus vehicle–vehicle or vehicle; and ^#^*P*<0.05, ^##^*P*<0.01 versus vehicle–DHK (Bonferroni *post hoc* test). Microdialysis data are expressed as percentages of four basal values. The arrow represents the start of the microinfusion with vehicle or s-AMPA (**h**–**k**). AMPA-R, AMPA receptor; DHK, dihydrokainic acid; FST, forced swimming test; IL, infralimbic cortex; NBQX, 2,3-dioxo-6-nitro-1,2,3,4-tetrahydrobenzo[f]quinoxaline-7-sulfonamide disodium salt; NSFT, novelty-suppressed feeding test; PrL, prelimbic cortex; VEH, vehicle.

Pretreatment with IL NBQX (10 nmoles) prevented the antidepressant-like response of IL DHK on the FST (Figure 3a). One-way ANOVA showed a significant effect of treatment on immobility (F_3,23_=4.117; *P*<0.05) and swimming behavior (F_3,23_=7.448; *P*<0.01). *Post hoc* comparisons showed that IL NBQX prevented the reduction of immobility (*P*<0.05) and the increased swimming behavior (*P*<0.01) of IL DHK (5 nmoles). Nonsignificant responses were observed for the control and NBQX groups.

### s-AMPA microinfusion in IL, but not PrL, induces antidepressant responses on the FST and NSFT

IL s-AMPA (50 pmoles per side) microinfusion produced an antidepressant-like response on the FST, with a significant decrease of immobility (*t*_(14)_=2.900; *P*<0.05) and increased swimming behavior (*t*_(14)_=3.123; *P*<0.01; Figure 3b). IL s-AMPA also evoked an antidepressant-like response on the NSFT (Figure 3). Thus, Kaplan–Meier survival analysis showed a significant difference between s-AMPA and vehicle groups (*t*_(19)_=2.127; *P*<0.05). Likewise, IL s-AMPA reduced the latency to eat compared with vehicle (*P*<0.05, unpaired *t*-test; Figure 3e). There were no differences in homecage food intake between IL s-AMPA and vehicle groups neither in the 5 min test (data in grams; vehicle: 0.65±0.08, s-AMPA: 0.43±0.09; *t*_*(19)*_=*1.824;* NS) nor in the 24 h test (vehicle: 28.20±0.62, s-AMPA: 26.38±2.28; *t*_(17)_=0.807; NS).

In contrast, PrL s-AMPA produced no significant behavioral response on the FST (Figure 3c) and NSFT (Figures 3f and g).

### IL and PrL s-AMPA effects on glutamate and 5-HT output

Baseline extracellular glutamate concentrations (pmol per fraction) in IL and PrL were 9.8±0.9 (*n*=20) and 10.0±0.9 (*n*=14), respectively (NS). s-AMPA perfusion enhanced the extracellular glutamate similarly in IL and PrL, with a significant effect of treatment (F_1,19_=19.480; *P*<0.001), fraction (F_8,152_=9.750; *P*<0.0001) and treatment × fraction interaction (F_8,152_=7.389; *P*<0.0001; IL data; Figure 3h, Supplementary Figure S2a). For PrL data, there was a significant effect of treatment (F_1,13_=11.590; *P*<0.01), fraction (F_8,104_=4.961; *P*<0.0001) and treatment × fraction interaction (F_8,104_=2.151; *P*<0.05; Figure 3i, Supplementary Figure S2b).

Baseline extracellular 5-HT concentrations (fmol per fraction) in IL and PrL were 5.0±1.1 (*n*=15) and 3.3±0.5 (*n*=12), respectively (NS). The perfusion of DHK into IL markedly increased 5-HT release, with a significant effect of treatment (F_1,13_=10.560; *P*<0.01), fraction (F_8,104_=5.416; *P*<0.0001) and treatment × fraction interaction (F_8,104_=5.638; *P*<0.0001; Figure 3j, Supplementary Figure S2c). Conversely, s-AMPA into PrL cortex did not change extracellular 5-HT with no significant effect of treatment, fraction or interaction in the two-way ANOVA (Figure 3k, Supplementary Figure S2d).

### Prevention of antidepressant responses of IL DHK by pCPA

**Figure 4:**
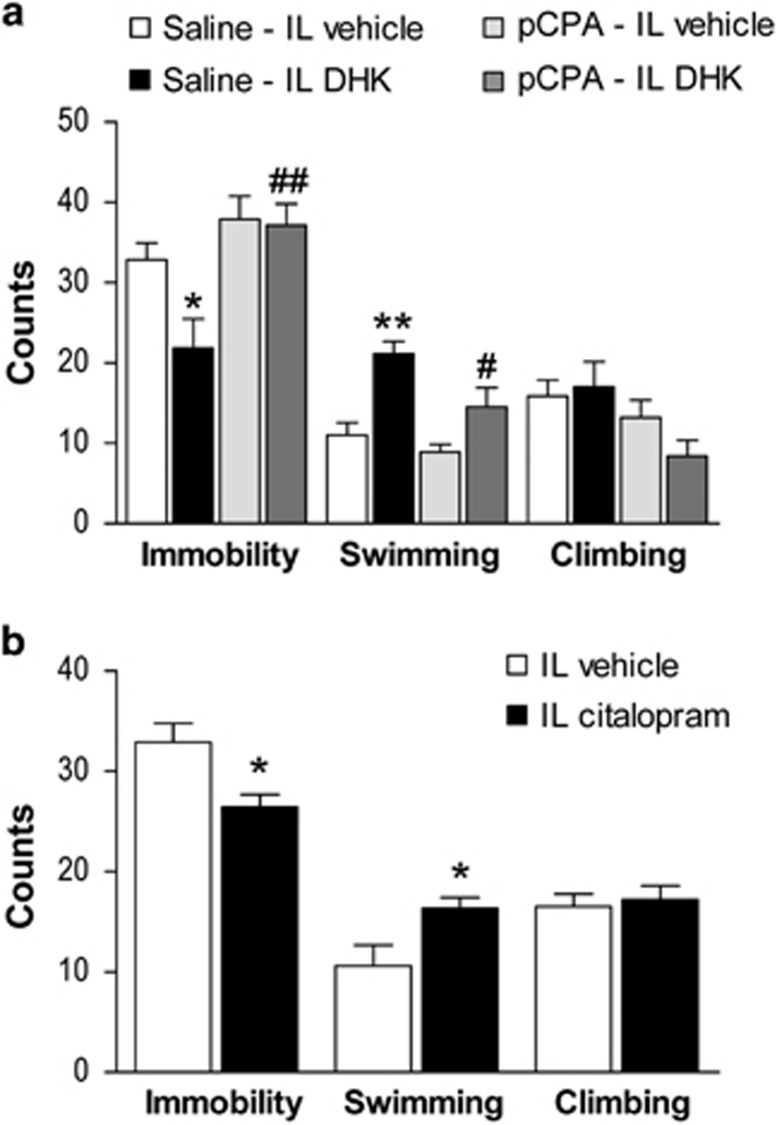
Serotonin is necessary for the DHK-induced antidepressant-like actions. (**a**) Effect of systemic pCPA (86 mg kg^−1^, 4 days) and IL DHK (5 nmoles) microinfusion on the FST. The reduction in immobility and increase in swimming counts observed in saline–IL DHK group compared with saline–IL vehicle (*n*=7, both groups) was prevented by previous intraperitoneal treatment with pCPA (pCPA–IL DHK; *n*=8). pCPA–IL vehicle group (*n*=9) was similar to controls. (**b**) The bilateral microinfusion of citalopram (15 pmoles) into IL produced a rapid antidepressant-like response, as shown by decreased immobility and increased swimming counts (IL vehicle, *n*=10; IL s-AMPA, *n*=9). The values are expressed as mean±s.e.m. *P<0.05, **P<0.01 versus saline–vehicle or vehicle; and ^#^*P*<0.05, ^##^*P*<0.01 versus saline–DHK (Bonferroni *post hoc* test). DHK, dihydrokainic acid; FST, forced swimming test; IL, infralimbic cortex; pCPA, 4-chloro-dl-phenylalanine methyl ester hydrochloride.

5-HT depletion by pCPA (86 mg kg^−1^, 4 days, intraperitoneally) prevented the antidepressant-like responses of IL DHK on the FST (Figure 4a). One-way ANOVA showed a significant effect of treatment for immobility (F_3,30_=6.380; *P*<0.01) and swimming behavior (F_3,30_=9.981; *P*<0.01) with significant *post hoc* differences in immobility (*P*<0.01) and swimming behavior (*P*<0.05) between pCPA-pretreated and saline-pretreated rats infused with IL DHK, indicating a full suppression of DHK effects in 5-HT-depleted rats. Nonsignificant differences were observed between the control and pCPA-treated rats.

Detection of neurotransmitter concentration in tissue samples showed that pretreatment with pCPA significantly decreased the concentration of 5-HT and its metabolite 5-hydroxyindoleacetic acid (5-HIIA) by 95% and 98% in the mPFC; and by 83% and 80% in the DR, respectively (*P*<0.01, all cases, data not shown).

### IL citalopram produces antidepressant responses on the FST

As results with pCPA indicated an involvement of serotonin in the antidepressant-like effects of DHK microinfusion, we examined the effects of IL microinfusion of the selective serotonin reuptake inhibitor citalopram. Thus, IL citalopram (15 pmoles per side) produced an antidepressant-like response on the FST (Figure 4b). Unpaired *t*-test showed that IL citalopram produced a significant decrease in immobility (*t*_(17)_=2.803; *P*<0.05) and an increase in swimming behavior (*t*_(17)_=2.377; *P*<0.05).

### *c-fos* mRNA expression after IL and PrL DHK or s-AMPA

As in previous studies,^36, 38, 39^ we used *c-fos* mRNA expression as a surrogate marker of neuronal activation to examine brain areas engaged in the antidepressant-like effects of DHK and s-AMPA microinfusion in IL.

**Figure 5:**
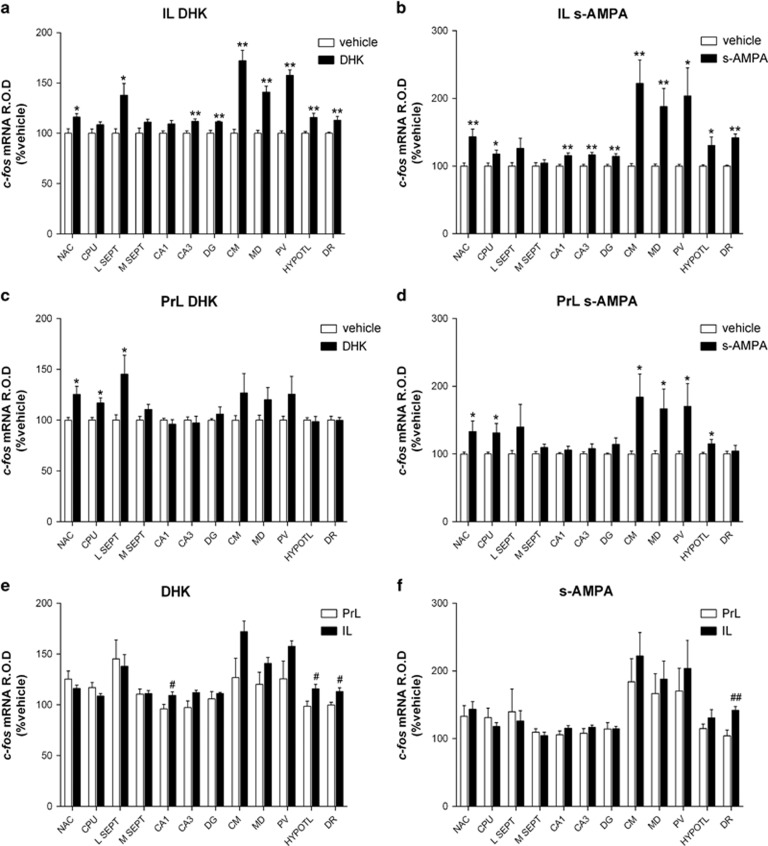
*c-fos* mRNA expression after bilateral IL and PrL DHK (5 nmoles) and s-AMPA (50 pmoles) microinfusion. Rats were killed 1 h after drug or vehicle microinfusion. (**a**–**f**) Bar graphs showing mean±s.e.m. of optical activity (arbitrary units) after treatments (*n*=6 per group), in different brain areas: CA1 and CA3, CA regions of the hippocampus; CM, centromedial nucleus of thalamus; CPU, caudate-putamen nuclei; DG, dentate gyrus; DHK, dihydrokainic acid; DR, dorsal raphe; HYPOTL, hypothalamus; IL, infralimbic cortex; L SEPT, lateral septum; MD, mediodorsal nucleus of the thalamus; M Sept, medial septum; NAC, nucleus accumbens; PrL, prelimbic cortex; PV, paraventricular nucleus. *P<0.05, **P<0.01 versus vehicle; ^#^*P*<0.05, ^##^*P*<0.01 versus PrL (Student's *t*-test).

IL DHK significantly increased *c-fos* expression in the nucleus accumbens and lateral septum (*P*<0.05), and in the CA3 and dentate gyrus region of the hippocampus, the centromedial, mediodorsal and paraventricular nuclei of the thalamus, the hypothalamus and the DR (*P*<0.01; Figure 5a). PrL DHK increased *c-fos* expression in the nucleus accumbens, caudate-putamen and lateral septum (*P*<0.05; Figure 5c). Significant differences between IL and PrL DHK were found in the CA1, hypothalamus and DR (*P*<0.05; Figure 5e). Likewise, IL s-AMPA significantly increased *c-fos* expression in the caudate-putamen, paraventricular and hypothalamus (*P*<0.05), and in the nucleus accumbens, CA1, CA3, dentate gyrus, centromedial, mediodorsal and DR (*P*<0.01; Figure 5b). PrL s-AMPA increased *c-fos* expression in the nucleus accumbens, caudate-putamen, centromedial, mediodorsal and paraventricular (*P*<0.05; Figure 5d). Significant differences between IL and PrL s-AMPA were found in the DR (*P*<0.01; Figure 5f).

### IL veratridine, DHK and s-AMPA effects on locomotor activity

Behavioral changes observed on the FST were not due to a drug-induced increase in general locomotor activity. On the contrary, unpaired *t*-test showed that IL veratridine (50 pmoles) reduced distance moved compared with vehicle group. No changes in locomotor activity were observed after IL DHK or s-AMPA microinfusion (Supplementary Figure S3).

## Discussion

The present study shows that the regionally selective enhancement of glutamatergic neurotransmission in the IL -but not PrL- induces robust antidepressant-like responses in rats. This effect appears to involve the activation of IL AMPA-R and the subsequent enhancement of serotonergic activity, as suggested by the large increase of 5-HT release associated to DHK and s-AMPA infusion in IL and by the prevention of antidepressant-like effects by 5-HT depletion with pCPA. Likewise, the involvement of 5-HT is also supported by the antidepressant-like response evoked by IL citalopram. Overall, our data emphasize the relevance of excitatory and serotoninergic neurotransmission in ventral mPFC on mood regulation.

First, we observed that veratridine, which is a non-selective depolarizing agent, evoked antidepressant-like responses when microinfused specifically into IL. Although veratridine is not neurotransmitter-selective, more than 80% of PFC neurons are excitatory and the PFC contains a dense network of afferent and efferent glutamatergic fibers that would release glutamate after veratridine microinfusion. In agreement, the optogenetic activation of IL pyramidal neurons evoked rapid antidepressant-like responses in rats.^21^ Then, we used a glutamate transporter inhibitor (DHK) to increase PFC glutamate. DHK selectively blocks the GLT-1, mainly localized in astrocytes and responsible for more than the 90% of the cortical glutamate uptake.^24, 25^ Similar to veratridine, IL -but not PrL- DHK induced antidepressant-like responses on the FST at the dose of 5 nmoles, whereas lower doses (1.5 nmoles) were ineffective. In addition, a 10-fold lower dose of DHK (0.15 nmoles) resulted without consequences on the FST. Although the present data suggest that antidepressant-like effects are mediated by an increase of excitatory neurotransmission, we cannot rule out that the excess glutamate may actually inhibit excitatory neurotransmission through depolarization blockade. Interestingly, IL DHK also evoked antidepressant-like responses on the NSFT. This test is sensitive to chronic, but not acute, classic antidepressants,^37^ as well as to systemic or IL ketamine.^21, 40^ Thus, the local administration of DHK into IL mimics the rapid effects of ketamine in this test.

Our results are consistent with the distinct -sometimes opposed- roles that IL and PrL have in the control of emotional signals.^17, 19, 41^ Recently, the intra-PFC infusion of DHK induced an anhedonic behavior in rats.^42^ However, it remains to be determined whether IL and PrL contribute differently to this phenotype.

The regional selectivity observed in the present study is in agreement with recent data indicating that DBS and ketamine exert antidepressant effects via their actions into the IL.^20, 21, 43^ Moreover, both treatments increase glutamate release in PFC^20, 22^ and involve the AMPA-R in their antidepressant effects.^20, 23^ Conversely, decreased levels of prefrontal AMPA-R subunits were observed in subjects with mood disorders^44^ and in rats after repeated stress.^45^ In line with these observations, we show that (i) the IL microinfusion of the AMPA-R antagonist NBQX blocked the antidepressant-like responses of IL DHK and (ii) IL s-AMPA evoked antidepressant-like responses. To our knowledge, this is the first study directly investigating the role of AMPA-R activation selectively in IL and PrL on depressive behaviors and provides further support for a crucial role of IL AMPA-R activation in the resilience to stress.

DHK and s-AMPA increased extracellular glutamate similarly in the IL and PrL, indicating that the behavioral differences were not due to a differential enhancement of glutamatergic neurotransmission in both regions. Interestingly, local DHK and s-AMPA elevated extracellular 5-HT when applied in IL, and slightly reduced it or left it unaffected when applied in PrL. Thus, we hypothesized that 5-HT could be involved in the antidepressant-like responses after enhancing IL glutamate. In support of this view, 5-HT depletion with pCPA prevented the antidepressant effects of IL DHK. Conversely, serotonin transporter blockade with citalopram in IL evoked antidepressant-like effects. This is the first observation that an intracerebral selective serotonin reuptake inhibitor application can evoke antidepressant effects similar to a systemic selective serotonin reuptake inhibitor. These observations are in line with the positive role that 5-HT had in the control of stress and suggest that the enhanced 5-HT release contributes to DHK-induced effects. Moreover, the increased swimming behavior observed in the FST is consistent with an antidepressant-mediated increase in serotonergic neurotransmission as previously reported.^33, 46^ This view is also supported by previous observations showing an association between enhanced 5-HT release and antidepressant-like responses after reducing the expression of 5-HT_1A_ autoreceptors,^47^ DBS^48, 49^ and ketamine administration.^50^

Although we cannot exclude a local effect of glutamate on 5-HT terminals in the mPFC, there are more data supporting the circuitry-based hypothesis. There is compelling evidence of reciprocal connectivity between PFC and DR.^27, 51, 52, 53^ Pyramidal neurons in the mPFC exert a tight control over brainstem monoaminergic neurons, including raphe 5-HT neurons.^26, 54^ The latter pathway controls animal response to behavioral challenges, as shown by the antidepressant-like responses after optogenetic stimulation of mPFC-DR projection.^55^ Hence, Fukumoto *et al.*^50^ showed that the antidepressant effects of PFC microinfusion of ketamine and mGlu2/3 receptor antagonism require the activation of raphe 5-HT neurons. Reciprocally, raphe 5-HT neurons control the activity of PFC pyramidal neurons.^28, 56^ Therefore, the differential 5-HT response in IL and PrL to the similar enhancement of glutamate in both mPFC subdivisions likely reflects a differential control of raphe 5-HT neurons by IL and PrL. Supporting this view, we have observed a differential expression of *c-fos* in the DR after DHK and s-AMPA infusion in IL and PrL, suggesting an increased activity in DR after IL drug treatments. Anatomical studies may support our results,^27^ yet it remains to be established by electrophysiological and imaging techniques. Hence, previous electrophysiological studies had shown that mPFC pyramidal neurons may activate or inhibit DR serotonin neurons.^26^ Excitations are monosynaptic and involve the activation of AMPA-R and *N*-methyl-d-aspartate receptor on serotonin neurons, whereas inhibitions are mediated by two different processes, (i) a local negative feedback involving 5-HT_1A_ autoreceptors and (ii) the activation of GABA interneurons in the midbrain raphe by mPFC inputs and the subsequent inhibition of serotonin neurons.^26, 57^ However, to date, it is uncertain whether IL and PrL inputs to the midbrain raphe may elicit selective responses, as suggested by the present data. In addition, IL and PrL are reciprocally connected and IL exerts an inhibitory control over PrL activity, which adds an additional element of complexity.^58^ Recent findings suggest that the activation of neuronal population in IL and its descending axons is implicated on the antidepressant-like responses of IL DBS, leading to an increased firing of raphe 5-HT neurons.^43^ Thus, an enhanced excitatory transmission in mPFC-raphe descending pathways^55^ could be a common feature of different fast-acting antidepressant strategies.

Finally, it has been described that an increase of presynaptic neuronal activity in the cerebral cortex slows the clearance of glutamate by astrocytes, increasing the persistence of glutamate in the extracellular space, which may facilitate receptor activation. This physiological mechanism represents a novel form of neuronal-astrocyte communication, challenging the previous view that glutamate transport by EAATs has solely a housekeeping function.^59^ In our studies with DHK, the reduction in glutamate transport facilitates glutamate availability and AMPA-R activation, which produce an antidepressant phenotype. Thus, given the role of glial cells in synaptic transmission,^60, 61^ further work is necessary to elucidate the role of the tripartite glutamate synapse in the pathophysiology of major depressive disorder.^62^

Overall, we show that the increase of glutamatergic tone in IL -but not PrL- exerts immediate antidepressant-like responses in rats. These effects are dependent on stimulation of AMPA-R and involve an increased serotonergic function. This adds to recent studies in the field supporting an important role of IL mPFC subdivision in the mechanism of action of fast-acting antidepressant strategies.
