# Supplementary material for: Glial GLT-1 blockade in infralimbic cortex as a new strategy to evoke rapid antidepressant-like effects in rats
Source: Transl Psychiatry. 2017 Feb 21;7(2):e1038–. doi: 10.1038/tp.2017.7 (PMC5438036; doi:10.1038/tp.2017.7)
Supplement: Supplementary Information [file tp20177x1.docx]

**Supplementary Legends**

**Figure S1.** Mean ± SEM of extracellular levels of glutamate and serotonin in IL **(a,c)** and PrL **(b,d)** cortex expressed as AUC from four dialysate fractions at basal conditions (aCSF) and during vehicle, 3 mM DHK nM, and 10 mM DHK perfusion. **p* < 0.05, ***p* < 0.01 *versus* vehicle (VEH) (Student’s *t*-test).

**Figure S2.** Mean ± SEM of extracellular levels of glutamate and serotonin in IL **(a, c)** and PrL **(b, c)** cortex expressed as AUC from four dialysate fractions at basal conditions (aCSF) and during vehicle or 100 µM s-AMPA perfusion. **p* < 0.05, ***p* < 0.01 *versus* vehicle (VEH) (Student’s *t*-test).

**Figure S3.** Mean ± SEM of distance moved (cm) by rats in an open field after bilateral IL vehicle, veratridine, DHK or s-AMPA infusion. Recordings were performed 10 min after drug infusion and lasted 15 min. ***p* < 0.01 *versus* vehicle (VEH) (Student’s *t*-test).
