# Supplementary figures and images for: Glial GLT-1 blockade in infralimbic cortex as a new strategy to evoke rapid antidepressant-like effects in rats
Source: Transl Psychiatry. 2017 Feb 21;7(2):e1038–. doi: 10.1038/tp.2017.7 (PMC5438036; doi:10.1038/tp.2017.7)

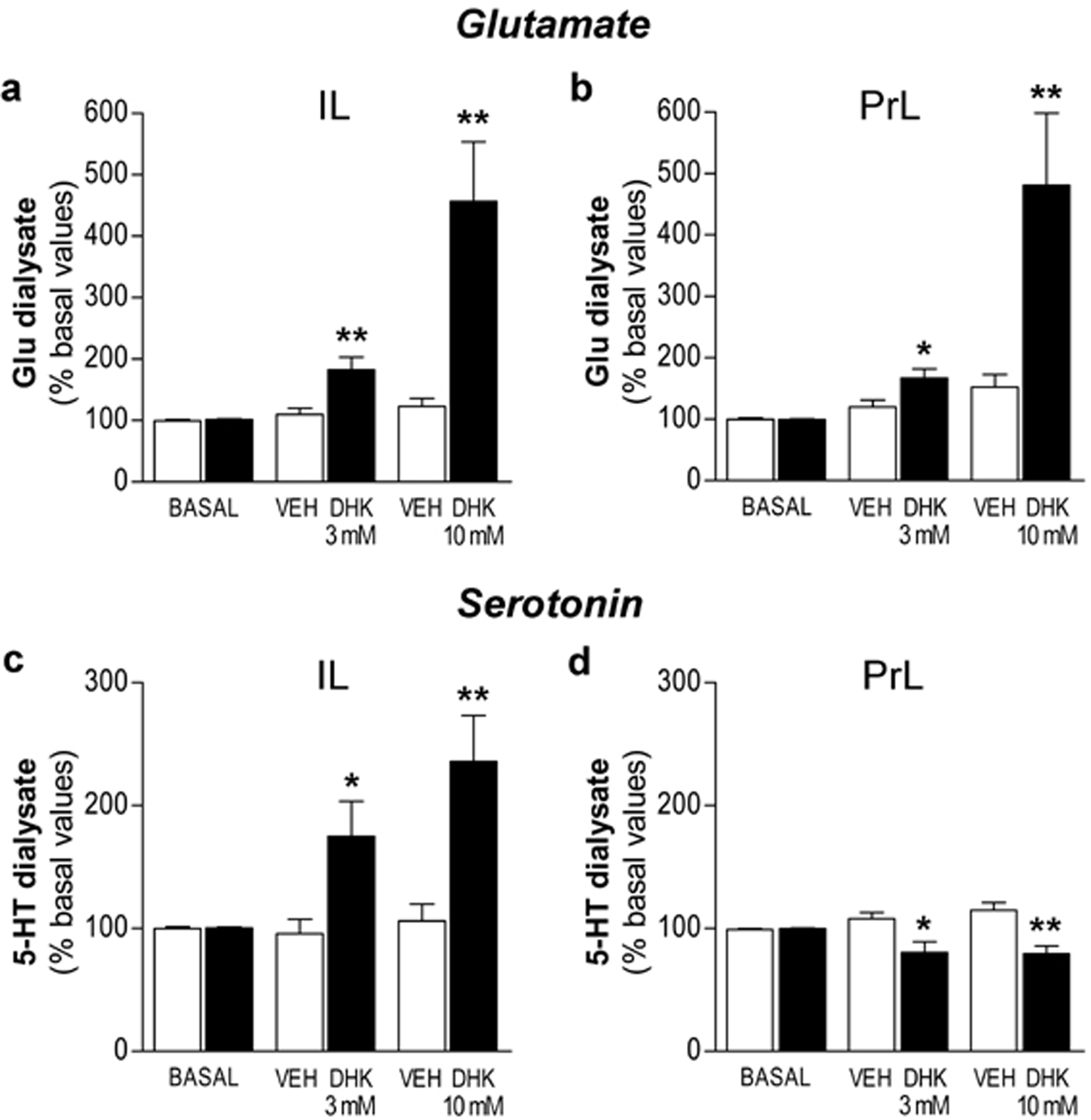

Supplement: Supplementary Figure 1 [file tp20177x2.tif]

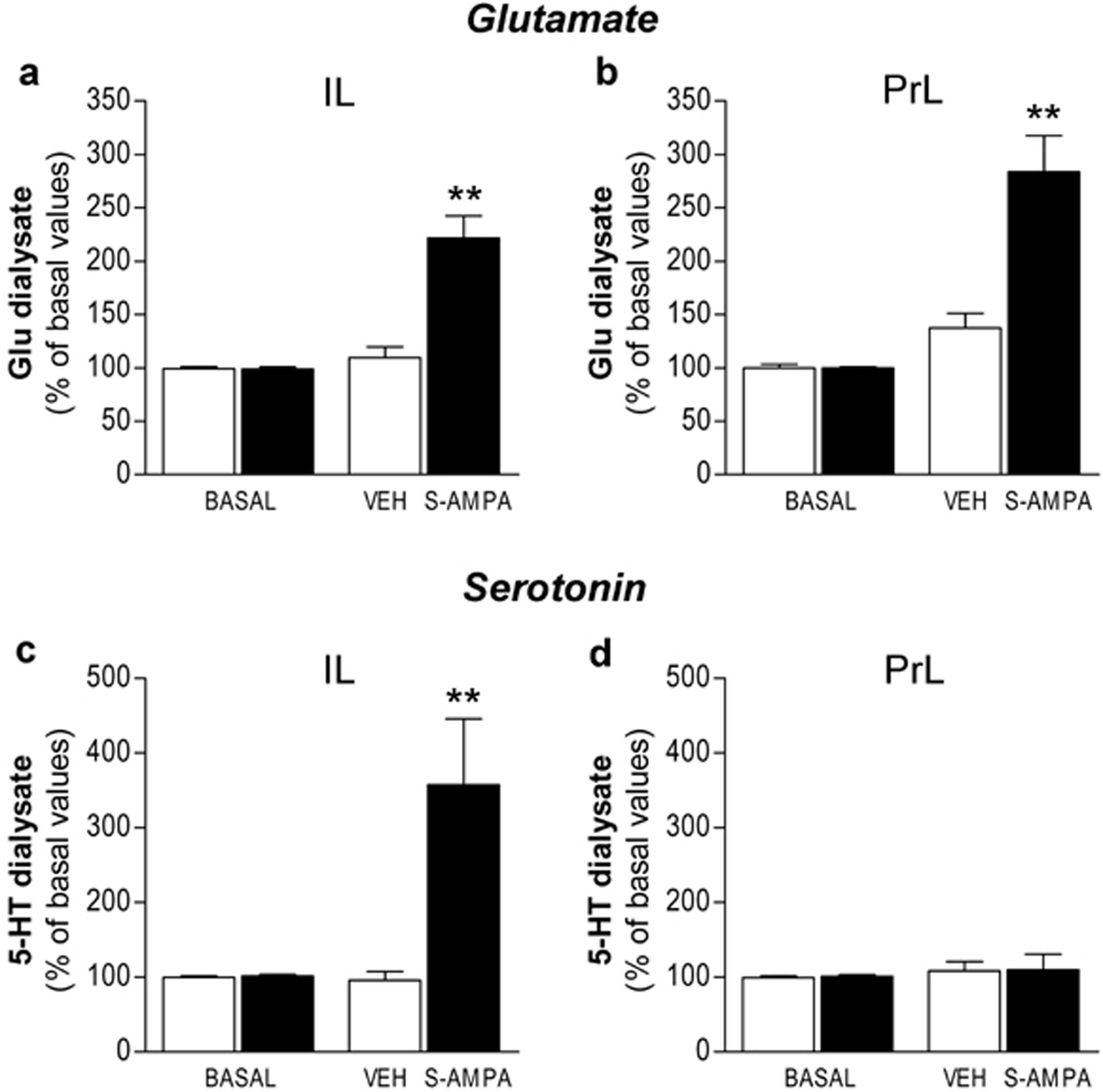

Supplement: Supplementary Figure 2 [file tp20177x3.tif]
